# Risk Stratification for Diabetic Kidney Disease Progression in Predicting All‐Cause and Cardiovascular Mortality in Type 2 Diabetes: A National Cohort Study From NHANES 1999–2018

**DOI:** 10.1155/jdr/9563077

**Published:** 2026-06-04

**Authors:** Ying Qiu, Jun Jiang, Hongqiang Zhang, Jianping Weng, Lei Lan

**Affiliations:** ^1^ Department of Nephrology, The First Affiliated Hospital of USTC, Division of Life Sciences and Medicine, University of Science and Technology of China, Hefei, Anhui, China, ustc.edu.cn; ^2^ Cheeloo College of Medicine, Shandong University, Jinan, Shandong, China, sdu.edu.cn; ^3^ Department of Endocrinology, The First Affiliated Hospital of USTC, Division of Life Sciences and Medicine, University of Science and Technology of China, Hefei, Anhui, China, ustc.edu.cn

**Keywords:** diabetic kidney disease, mortality, risk stratification, Type 2 diabetes

## Abstract

Diabetic kidney disease (DKD) substantially contributes to premature mortality in individuals with Type 2 diabetes (T2D). Although the 2024 American Diabetes Association (ADA) guideline incorporates the risk stratification of DKD progression into routine diabetes care, its predictive validity for mortality remains underexplored in real‐world populations. Using data from 6936 adults with T2D enrolled in the US National Health and Nutrition Examination Survey (NHANES) from 1999 to 2018, this study examined whether baseline DKD progression risk categories—based on estimated glomerular filtration rate (eGFR) and urine albumin‐to‐creatinine ratio (UACR)—can predict all‐cause and cardiovascular mortality. Risk of DKD progression was classified as low, moderately increased, high, or very high. Mortality outcomes were identified via linkage to the National Death Index until December 31, 2019. Cox proportional hazards models were used to estimate hazard ratios (HRs), adjusting for demographics, comorbidities, lifestyle behaviors, laboratory values, and medication use. Over a median follow‐up of 98 months, 1781 all‐cause and 623 cardiovascular deaths occurred. Compared with the low‐risk group, the very high‐risk group had significantly higher risks of all‐cause mortality (adjusted HR: 2.53; 95% CI: 2.15–2.98) and cardiovascular mortality (adjusted HR: 2.47; 95% CI: 1.87–3.27), with a clear dose‐response gradient across risk categories (*p* for trend < 0.001). Notably, this graded relationship remained stable across most examined subgroups, and significant interactions were observed specifically among individuals aged < 65 years and those using renin–angiotensin system (RAS) inhibitors. These findings confirm the robust prognostic utility of the ADA‐endorsed risk stratification matrix of DKD progression for mortality in T2D and highlight its potential to inform risk‐based individualized management strategies in clinical practice. This study also provides pragmatic evidence supporting the implementation of guideline recommendations.

## 1. Introduction

Global diabetes prevalence is increasing [1]. Approximately 40% of affected individuals develop diabetic kidney disease (DKD), which substantially elevates cardiovascular risk and mortality [2, 3]. This highlights the critical need for early risk stratification and management of kidney disease progression in patients with diabetes.

Glomerular filtration rate (GFR) and urine albumin‐to‐creatinine ratio (UACR) are two key indicators for assessing kidney function status [4, 5]. Previous large‐scale meta‐analyses have confirmed that, regardless of whether in the general population [6–8], high‐risk populations (including patients with hypertension, diabetes, and cardiovascular disease [CVD]) [6, 9], or in chronic kidney disease (CKD) patients [6, 10], increased UACR and decreased GFR are independently associated with higher risks of CKD progression, end‐stage renal disease (ESRD), CVD events, and mortality, and the combination of these two indicators further amplifies the risk. Consequently, based on this evidence, the 2012 Kidney Disease Improving Global Prognosis Organization (KDIGO) guidelines first proposed a risk stratification system categorizing CKD patients into low, moderately increased, high, and very high‐risk groups [11]. This classification utilizes a combination of GFR and UACR to predict risks of kidney disease progression, cardiovascular events, and mortality.

However, studies and large‐scale meta‐analyses underpinning the guideline primarily utilized single measures of GFR or UACR, or their combination, to assess CKD progression and prognosis. Subsequent studies, such as the Chronic Renal Insufficiency Cohort (CRIC) study [12, 13], the Systolic Blood Pressure Intervention Trial (SPRINT) [14], and analyses of CKD patients in the National Health and Nutrition Examination Survey (NHANES) database [15], also mainly focused on the prognostic value of estimated glomerular filtration rate (eGFR) and UACR—either alone, as continuous variables, or through complex combinations—rather than on clinically applicable risk stratification systems. Recently, a single‐center study developed a nomogram to predict renal survival in biopsy‐proven DKD patients using eGFR, common logarithm of UACR, cystatin C, hemoglobin, and fibrinogen [16]. However, that study was limited to a small biopsy cohort and did not validate the risk stratification recommended by the American Diabetes Association (ADA) in a general population with Type 2 diabetes (T2D). In addition, another large international meta‐analysis assessed the associations between eGFR (based on creatinine or creatinine plus cystatin C), UACR, and the risk of 10 adverse outcomes (including mortality, cardiovascular events, and kidney failure) across a broad population spectrum encompassing the general population, high‐risk cardiovascular groups, and patients with CKD [17]. However, this study also did not classify individuals into “low,” “moderate,” “high,” or “very high” risk categories based on the KDIGO framework. Instead, it mainly emphasized the clinical applicability of a new eGFR equation that uses serum cystatin C and does not include race.

As evidence continues to accumulate, the ADA and KDIGO jointly issued a consensus report in 2022 [18]. This report formally integrated the risk stratification heat map of CKD progression into diabetes management systems for the first time. It mandates annual screening for all individuals with diabetes based on eGFR and UACR measurements. Furthermore, it advocates for differential management of DKD patients at varying risk strata regarding frequency of visits, preventative interventions, and referral to nephrology, enhancing its role in clinical decision‐making. By the 2024 ADA standards of care in diabetes, the risk stratification framework of CKD progression further evolved and was strengthened [19]. This iteration not only retained the risk stratification heat map as the core risk assessment tool but also explicitly linked it to pharmacotherapy interventions and patient education pathways.

However, evidence on the practical utility of guideline‐recommended risk stratification in clinical practice remains limited. Existing studies have primarily examined the association of risk categories of CKD progression with cardiovascular risk factors, such as the prevalence and management rates of hypertension, diabetes, and dyslipidemia, rather than mortality outcomes [20]. Others have studied the relationship between risk stratification and progression through risk categories with renal function decline, CVD, and mortality, predominantly in nondiabetic cohorts [21, 22]. Critically, no study has validated the predictive value of this risk stratification for mortality in real‐world populations with diabetes, creating a crucial evidence gap for the clinical implementation of current guidelines.

Therefore, utilizing data from NHANES spanning 1999 to 2018, we aim to validate the predictive value of the 2024 ADA‐based risk stratification matrix of DKD progression for both all‐cause mortality and cardiovascular mortality among US T2D adults. This research will be conducted within the multiethnic US population to provide pragmatic evidence supporting the implementation of guideline recommendations.

## 2. Materials and Methods

### 2.1. Study Population

NHANES is a national survey that measures the health and nutrition of adults and children in the United States. It employs a stratified, multistage probability sampling design, and the sample is nationally representative. Data are collected through structured interviews conducted in participants′ homes, health examinations at mobile examination centers, and laboratory analysis of specimens [23]. The survey protocol was approved by the Institutional Review Board of the National Center for Health Statistics (NCHS), and all participants provided written informed consent. Additionally, this study was also reviewed and approved by the Ethics Committee of the First Affiliated Hospital of the University of Science and Technology of China (ID: 2026‐ky180).

**Figure 1:**
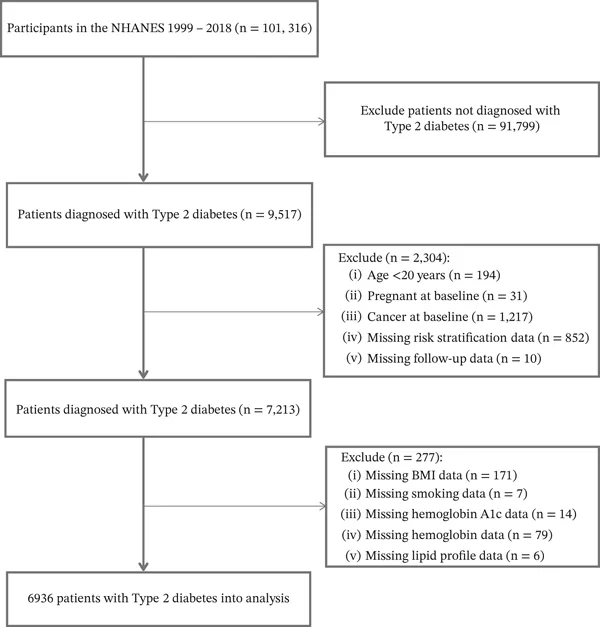
Flow chart for inclusion and exclusion of the study population. HNAHES, National Health and Nutrition Examination Survey; BMI, body mass index.

We utilized data from NHANES 1999–2018, including participants aged 20 years and older diagnosed with T2D. T2D was defined as self‐reported physician diagnosis, use of insulin or oral hypoglycemic agents, fasting blood glucose levels ≥ 7.0 mmol/L (126 mg/dL), random blood glucose or 2‐h oral glucose tolerance test blood glucose ≥ 11.1 mmol/L (200 mg/dL), or hemoglobin A1c (HbA1c) ≥ 6.5% [24]. Participants were excluded based on the following criteria: (1) age under 20; (2) pregnancy at baseline; (3) baseline cancer diagnosis; (4) missing baseline risk stratification data; (5) missing follow‐up data; and (6) missing important baseline clinical measurements. Ultimately, this study included 6936 patients with T2D (Figure 1).

### 2.2. Risk Stratification of DKD Progression

According to eGFR and albuminuria, the risk of DKD progression was categorized as low risk (if no other markers of kidney disease, no CKD), moderately increased risk, high risk, and very high risk [11, 18, 19]. We calculated eGFR based on serum creatinine, gender, and age, without race, using the 2021 CKD‐EPI formula [25]. Albuminuria was assessed by UACR, which was derived by dividing the concentration of urinary albumin (mg/dL) by the concentration of urinary creatinine (mg/L).

### 2.3. Covariates

A comprehensive dataset was collected from the study participants, encompassing demographic details, physical examinations, laboratory blood tests, and medical histories. The demographic information included age, gender, race and ethnicity, education level, marital status, and the family income poverty rate (PIR). Race and ethnicity categories included Mexican American, non‐Hispanic White, non‐Hispanic Black, other Hispanic, and other race. Education levels were categorized as less than high school, high school or equivalent, and college and above. In addition, marital status was divided into married or living with a partner, widowed/divorced/separated, and never married. PIR was categorized as ≤ 1.3, 1.3–3.5, and > 3.5.

Standardized questionnaires were utilized to obtain information on smoking, alcohol consumption, diabetes duration, hypertension, CVD, stroke, and medication use—antihypertensive drugs, renin–angiotensin system (RAS) inhibitors, glucose‐lowering drugs (including insulin and oral glucose‐lowering drugs), and lipid‐lowering drugs. Smoking status was categorized as follows: never smoking, former smoking, and current smoking. Alcohol consumption status was recorded as follows: nondrinkers, low‐to‐moderate drinkers, and heavy drinkers. Hypertension was defined as having a self‐reported history of hypertension or use of antihypertensive medications or blood pressure readings of systolic blood pressure (SBP) ≥ 140 mmHg or diastolic blood pressure (DBP) ≥ 90 mmHg. History of CVD included self‐reported angina pectoris, congestive heart failure, coronary heart disease, and heart attack.

Blood pressure (including SBP and DBP), height, weight, and waist circumference were measured using standard methods at the NHANES Mobile Examination Center (MEC). Body mass index (BMI) was calculated as weight in kilograms divided by height in square meters. It is important to note that SBP and DBP do not represent conventional averages, and they were calculated according to the established protocol as specified in the analytic note [26].

Laboratory indicators were also collected, including baseline values for fasting triglycerides, total cholesterol (TC), HbA1c, serum albumin, hemoglobin, and serum uric acid (UA). Further details of these measurements were documented in the NHANES Laboratory Medical Technologists Procedures Manual [23].

### 2.4. Ascertainment of Mortality

The outcomes of this study were all‐cause and cardiovascular mortality [27]. All‐cause mortality was defined as death from any cause, whereas cardiovascular mortality included deaths attributed to heart diseases (ICD‐10 codes I00‐I09, I11, I13, I20‐I51) and cerebrovascular diseases (ICD‐10 codes I60‐I69), according to the International Classification of Diseases, 10th Clinical Modification (ICD‐10) system [28]. Mortality data from NHANES were linked to death certificate data from the National Death Index of the NCHS until December 31, 2019, employing a probability matching algorithm [29].

### 2.5. Statistical Analysis

The participants were categorized into the following groups according to their risk class: low risk, moderately increased risk, high risk, and very high risk. The results for continuous variables were presented as survey‐weighted means (95% confidence interval [CI]), with group differences evaluated by survey‐weighted linear regression. For categorical variables, the results were presented as survey‐weighted percentages (95% CI), with group differences evaluated by the survey‐weighted chi‐square test.

The probabilities of survival outcomes were calculated according to the Kaplan–Meier method and compared using the log‐rank test. Cox proportional hazard models were then used to compare the risks of all‐cause and cardiovascular mortality among study participants with different risk stratifications of DKD progression, after which a trend analysis was conducted. The association between mortality events and the risk stratification of DKD progression was then estimated by calculating the hazard ratio (HR) and 95% CI. The follow‐up time was computed from the NHANES MEC date to the date of mortality events, or December 31, 2019, whichever came first. The proportional hazard assumptions were examined using Schoenfeld residuals. Due to the extensive number of risk factors investigated in this study, only relevant demographic characteristics and traditional factors associated with the risk stratification of DKD progression and deaths were included in the multivariable Cox regression analysis. The selection of covariates was based on a combination of prior literature, clinical relevance, and potential confounding effects on the relationship between kidney function and mortality. Specifically, Model 1 was unadjusted. Model 2 was adjusted for basic demographic variables, including gender, age, race/ethnicity, family PIR, education level, and marital status, which are well‐established determinants of both kidney disease and mortality risk. Model 3 was further adjusted for alcohol consumption, smoking status, BMI, HbA1c, diabetes duration, history of hypertension, history of CVD, history of stroke, use of glucose‐lowering drugs, use of RAS inhibitors, and use of lipid‐lowering drugs based on Model 2. Model 4 made a comprehensive adjustment for potential confounders and further adjusted for hemoglobin, TC, triglycerides, albumin, and UA based on Model 3, to account for residual confounding.

In addition, we conducted stratified analyses by age (< 65 vs. ≥ 65 years), gender, BMI (< 30 vs. ≥ 30 kg/m^2^), baseline smoking status (never, former, and current), baseline HbA1c (< 7.0% vs. ≥ 7.0%), baseline TC (≤ 5.18 vs. >5.18 mmol/L), diagnosis of hyperuricemia and hypertension at baseline, use of glucose‐lowering drugs, use of RAS inhibitors, and use of lipid‐lowering drugs at baseline. Hyperuricemia was defined as a serum UA level above 420 umol/L in men and 360 umol/L in women [30]. In the subgroup analysis, the model was not adjusted for the stratification variable itself.

*p* < 0.05 was considered statistically significant in all analyses. All statistical analyses were executed using R software (Version 4.2.0) and EmpowerStats software (https://www.empowerstats.com/, X&Y solutions Inc., Boston, Massachusetts, United States).

## 3. Results

### 3.1. Baseline Characteristics

We analyzed 6936 individuals with T2D stratified into four DKD progression risk groups—low risk group (*n* = 4278), moderately increased risk group (*n* = 1574), high risk group (*n* = 638), and very high risk group (*n* = 446)—revealing significant demographic and clinical variations. Mean age progressively increased with DKD risk (low‐risk vs. very high‐risk: 55.6 vs. 69.4 years, *p* < 0.001). No significant differences were observed in gender distribution (*p* = 0.459) or overall BMI (*p* = 0.546); however, BMI category distribution differed significantly (*p* = 0.013), with a higher proportion of normal‐weight individuals in the very high‐risk group. Furthermore, racial and socioeconomic disparities were evident. The proportion of non‐Hispanic Black participants increased progressively with DKD risk (13.6%–24.5%), whereas non‐Hispanic White representation declined (61.5%–52.9%, *p* = 0.042). Participants in the very high‐risk group had lower household incomes (PIR ≤ 1.3, very high‐risk vs. low‐risk: 36.9% vs. 23.3%, *p* < 0.001) and lower educational attainment (college or above, very high‐risk vs. low‐risk: 32.4% vs. 51.4%, *p* < 0.001). Moreover, marital status showed significant variation, with widowed individuals comprising 43.6% of the very high‐risk group (low‐risk: 22.3%, *p* < 0.001). Regarding health behaviors, current smoking was less prevalent in the very high‐risk group (8.5%), whereas the proportion of nondrinkers increased markedly (47.1%, *p* < 0.001). Diabetes duration emerged as a critical progression marker: 70.3% of patients in the very high‐risk group had disease duration > 10 years, compared with 31.1% in the low‐risk group (*p* < 0.001). Comorbidity analysis revealed progressive increases in the prevalence of hypertension (69.3%–96.9%, *p* < 0.001), CVD (13.6%– 47.0%, *p* < 0.001), and stroke (4.5%–24.8%) across risk stratifications. Metabolic and cardiovascular parameters demonstrated significant variations across risk groups (all *p* < 0.001). In terms of prescription medications, use of antihypertensive and glucose‐lowering drugs at baseline increased progressively from the low‐risk to the very high‐risk group (respectively, 55.8%–88.3% and 50.4%–73.9%, both *p* < 0.001), whereas the use of RAS inhibitors decreased gradually across the same risk gradient (39.3%–11.3%, *p* < 0.001). SBP incrementally rose from 126.9 to 144.7 mmHg (Table 1).

**Table 1:** Baseline characteristics of the included patients with Type 2 diabetes by risk stratification of DKD progression.

Variables	Overall	Low risk group	Moderately increased risk group	High risk group	Very high risk group	*p* value
*N*	**6936**	**4278**	**1574**	**638**	**446**	
Age (years)	57.6 (56.9, 58.2)	55.6 (54.8, 56.4)	59.4 (58.1, 60.7)	63.3 (61.3, 65.4)	69.4 (67.4, 71.4)	< 0.001
Gender						0.459
Male	51.9 (49.8, 54.0)	52.0 (49.1, 54.8)	51.5 (46.5, 56.6)	55.8 (47.8, 63.5)	45.1 (36.6, 53.9)	
Female	48.1 (46.0, 50.2)	48.0 (45.2, 50.9)	48.5 (43.4, 53.5)	44.2 (36.5, 52.2)	54.9 (46.1, 63.4)	
BMI (kg/m^2^)	32.7 (32.3, 33.1)	32.7 (32.2, 33.2)	32.9 (32.1, 33.7)	32.6 (31.1, 34.1)	31.9 (30.8, 33.1)	0.546
BMI category (kg/m^2^)						0.013
≤ 25	13.3 (11.8, 15.1)	12.5 (10.8, 14.4)	13.2 (10.3, 16.9)	15.4 (10.4, 22.3)	23.3 (16.5, 31.8)	
25–≤ 30	28.5 (26.4, 30.6)	30.2 (27.5, 32.9)	24.8 (20.8, 29.2)	27.6 (21.3, 34.9)	21.0 (15.0, 28.6)	
> 30	58.2 (55.6, 60.7)	57.3 (54.2, 60.3)	62.0 (57.2, 66.6)	57.0 (48.9, 64.7)	55.7 (47.0, 64.0)	
Race/ethnicity						0.042
Mexican American	10.3 (8.7, 12.2)	10.0 (8.3, 11.9)	12.2 (9.9, 14.9)	10.0 (6.6, 14.9)	6.3 (3.6, 10.7)	
Other Hispanic	6.6 (5.3, 8.2)	6.5 (5.1, 8.2)	6.5 (4.5, 9.3)	8.5 (4.5, 15.3)	5.6 (3.3, 9.5)	
Non‐Hispanic White	60.0 (56.7,63.3)	61.5 (57.8, 65.0)	58.1 (52.7, 63.3)	56.9 (49.3, 64.2)	52.9 (43.5, 62.1)	
Non‐Hispanic Black	14.6 (12.8, 16.6)	13.6 (11.7, 15.6)	15.3 (12.5, 18.6)	16.2 (12.2, 21.2)	24.5 (18.3, 32.1)	
Other race	8.5 (7.1, 10.1)	8.5 (7.0, 10.3)	7.9 (5.6, 11.1)	8.4 (5.7, 12.4)	10.7 (6.2, 18.0)	
Family PIR						< 0.001
≤ 1.3	25.5 (23.6, 27.5)	23.3 (21.3, 25.4)	26.4 (22.4, 30.7)	36.2 (29.7, 43.2)	36.9 (29.3, 45.2)	
1.3–≤ 3.5	40.6 (37.9, 43.4)	39.2 (36.1, 42.4)	41.7 (36.6, 47.0)	49.6 (42.5, 56.7)	40.5 (31.2, 50.4)	
> 3.5	33.9 (31.0, 36.9)	37.5 (34.1, 41.0)	31.9 (26.7, 37.7)	14.2 (9.4, 21.0)	22.7 (14.9, 33.0)	
Education level						< 0.001
Less than high school	25.3 (23.3, 27.4)	22.4 (20.0, 25.0)	28.6 (24.9, 32.6)	36.7 (30.7, 43.0)	34.5 (27.1, 42.7)	
High school or equivalent	26.4 (24.2, 28.8)	26.2 (23.5, 29.2)	26.9 (21.8, 32.6)	23.5 (18.3, 29.6)	33.1 (24.2, 43.5)	
College or above	48.3 (45.6, 50.9)	51.4 (48.0, 54.7)	44.6 (38.8, 50.5)	39.8 (32.6, 47.6)	32.4 (24.6, 41.4)	
Marital status						< 0.001
Married or living with partner	63.9 (61.8, 66.1)	67.1 (64.3, 69.7)	62.1 (57.0, 66.9)	50.5 (41.7, 59.2)	48.5 (39.8, 57.2)	
Never married	9.9 (8.6, 11.5)	10.6 (9.0, 12.5)	7.7 (5.5, 10.6)	11.1 (6.7, 17.8)	7.9 (3.6, 16.8)	
Widowed	26.1 (24.2, 28.1)	22.3 (19.9, 24.9)	30.2 (26.1, 34.8)	38.5 (31.2, 46.3)	43.6 (35.7, 51.8)	
Smoking status						0.013
Never	49.6 (47.1, 52.0)	51.6 (48.4, 54.8)	43.3 (38.4, 48.2)	47.2 (40.3, 54.2)	53.0 (43.0, 62.7)	
Former	32.5 (30.2, 34.9)	30.6 (27.7, 33.7)	36.4 (31.9, 41.1)	35.1 (28.2, 42.7)	38.5 (29.5, 48.4)	
Current	17.9 (16.2, 19.8)	17.8 (15.7, 20.1)	20.4 (16.5, 24.9)	17.8 (12.6, 24.5)	8.5 (4.7, 14.9)	
Alcohol consumption						< 0.001
Nondrinker	19.4 (17.2, 21.8)	17.8 (15.5, 20.4)	17.6 (13.2, 23.1)	25.1 (17.4, 34.9)	47.1 (38.4, 56.0)	
Low‐to‐moderate drinker	41.4 (38.1, 44.8)	42.5 (38.8, 46.3)	41.6 (35.5, 48.0)	36.0 (27.7, 45.1)	30.6 (22.8, 39.7)	
Heavy drinker	39.2 (35.8, 42.7)	39.7 (35.7, 43.8)	40.8 (34.8, 47.1)	38.9 (30.9, 47.6)	22.3 (16.2, 29.9)	
Diabetes duration (years)						< 0.001
≤ 5	40.3 (37.3, 43.3)	46.5 (42.9, 50.1)	35.4 (29.7, 41.5)	22.3 (16.1, 30.1)	15.8 (10.2, 23.7)	
5–≤ 10	21.9 (19.3, 24.7)	22.4 (19.6, 25.5)	22.1 (16.6, 28.9)	22.7 (14.8, 33.2)	13.8 (8.2, 22.3)	
> 10	37.8 (35.1, 40.6)	31.1 (27.9, 34.5)	42.5 (36.5, 48.8)	54.9 (46.4, 63.2)	70.3 (61.2, 78.1)	
Comorbidities						
Hypertension	74.6 (72.4, 76.7)	69.3 (66.4, 72.0)	82.3 (77.2, 86.4)	88.2 (81.6, 92.6)	96.9 (85.9, 99.4)	< 0.001
CVD	17.6 (15.7, 19.7)	13.6 (11.4, 16.2)	20.2 (16.6, 24.4)	29.2 (23.7, 35.4)	47.0 (38.8, 55.4)	< 0.001
Stroke	6.6 (5.5, 7.9)	4.5 (3.4, 5.8)	6.5 (4.0, 10.3)	16.1 (11.2, 22.7)	24.8 (16.7, 35.1)	< 0.001
Prescription medications						
Antihypertensive drugs	60.8 (58.5, 63.0)	55.8 (53.0, 58.6)	66.2 (60.9, 71.1)	74.3 (66.5, 80.7)	88.3 (80.1, 93.4)	< 0.001
RAS inhibitors	34.6 (31.9, 37.3)	39.3 (35.6, 43.1)	33.1 (27.3, 39.6)	22.3 (15.5, 30.9)	11.3 (7.2, 17.3)	< 0.001
Glucose‐lowering drugs	53.7 (51.0, 56.3)	50.4 (47.0, 53.7)	56.6 (51.2, 61.8)	63.4 (54.5, 71.5)	73.9 (66.3, 80.4)	< 0.001
Lipid‐lowering drugs	44.8 (42.8, 46.8)	42.8 (40.2, 45.4)	46.9 (41.4, 52.4)	43.4 (36.6, 50.3)	68.7 (61.8, 74.9)	< 0.001
SBP (mmHg)	130.0 (129.1, 130.9)	126.9 (125.9, 127.9)	134.8 (132.8, 136.9)	135.5 (132.1, 139.0)	144.7 (140.7, 148.7)	< 0.001
DBP (mmHg)	70.5 (69.8, 71.3)	71.0 (70.1, 71.8)	71.3 (69.8, 72.7)	68.8 (65.9, 71.8)	63.0 (59.7, 66.3)	< 0.001
Hemoglobin (g/dL)	13.9 (13.8, 14.0)	13.9 (13.8, 14.0)	14.0 (13.8, 14.1)	13.8 (13.6, 14.1)	13.8 (13.5, 14.1)	0.669
HbA1c (%)	7.0 (7.0, 7.1)	6.9 (6.8, 7.0)	7.3 (7.1, 7.5)	7.7 (7.4, 8.0)	7.1 (6.8, 7.3)	< 0.001
Total cholesterol (mmol/L)	4.9 (4.9, 5.0)	4.9 (4.8, 5.0)	5.0 (4.9, 5.1)	5.1 (4.9, 5.3)	4.6 (4.4, 4.8)	0.009
Triglycerides (mmol/L)	2.0 (1.9, 2.1)	1.9 (1.8, 2.0)	2.2 (2.0, 2.5)	2.5 (2.1, 2.8)	1.8 (1.6, 1.9)	< 0.001
Albumin (g/L)	41.6 (41.4, 41.7)	41.9 (41.7, 42.1)	41.5 (41.2, 41.8)	40.0 (39.5, 40.5)	38.9 (38.1, 39.7)	< 0.001
Uric acid (umol/L)	347.6 (342.8, 352.5)	335.8 (330.5, 341.2)	349.9 (341.1, 358.7)	397.1 (381.1, 413.0)	434.1 (412.7, 455.4)	< 0.001
All‐cause mortality						< 0.001
Alive	78.5 (76.6, 80.3)	86.0 (83.8, 87.9)	69.4 (65.3, 73.2)	58.9 (52.2, 65.3)	40.3 (31.4, 49.9)	
Death	21.5 (19.7, 23.4)	14.0 (12.1, 16.2)	30.6 (26.8, 34.7)	41.1 (34.7, 47.8)	59.7 (50.1, 68.6)	
Cardiovascular mortality						< 0.001
Alive	92.1 (90.9, 93.1)	95.6 (94.5, 96.5)	87.4 (83.9, 90.3)	81.7 (75.9, 86.4)	77.8 (67.3, 85.6)	
Death	7.9 (6.9, 9.1)	4.4 (3.5, 5.5)	12.6 (9.7, 16.1)	18.3 (13.6, 24.1)	22.2 (14.4, 32.7)	

*Note:* Data in the table are presented as follows: For continuous variables, survey‐weighted mean (95% confidence interval [CI]) and *p* value were survey‐weighted by linear regression; for categorical variables, survey‐weighted percentage (95% CI) and *p* value were survey‐weighted by chi‐square test.

Abbreviations: BMI, body mass index; CVD, cardiovascular disease; DBP, diastolic blood pressure; DKD, diabetic kidney disease; HbA1c, hemoglobin A1c; PIR, poverty‐income ratio; SBP, systolic blood pressure.

### 3.2. Survival Analysis

**Figure 2:**
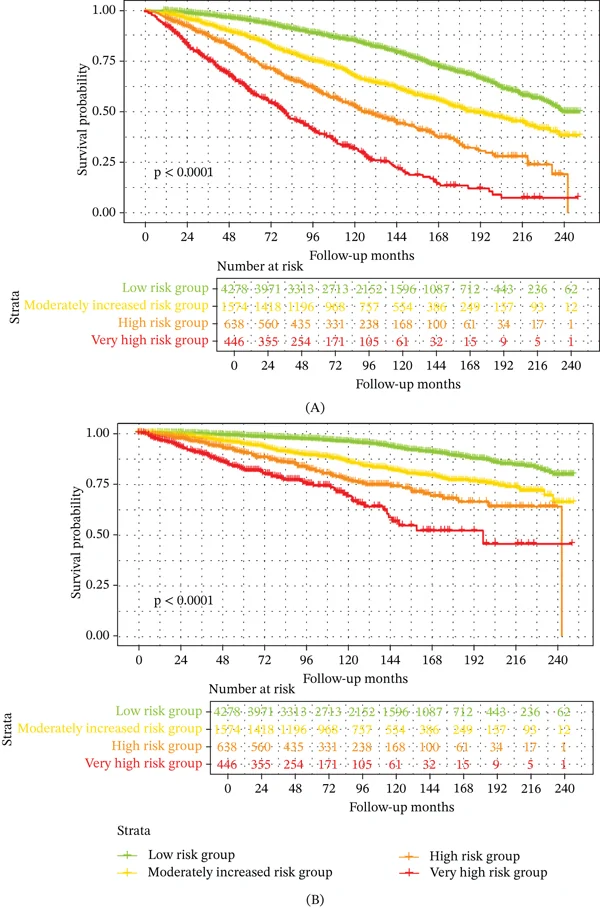
Time‐to‐survival probability distribution for (A) all‐cause mortality and (B) cardiovascular mortality in patients with Type 2 diabetes.

During a median follow‐up period of 98.0 months, 1781 cases of all‐cause mortality and 623 cases of cardiovascular mortality were documented. The time‐to‐survival probabilities distributions for all‐cause and cardiovascular mortality among individuals with T2D were examined, with these patients categorized into four distinct risk groups of DKD progression. The survival probability curves demonstrated a marked gradient, with the very high‐risk group exhibiting significantly lower survival probabilities compared with the low‐risk group (*p* < 0.001). During the follow‐up period, the number of patients at risk decreased substantially, particularly in the very high‐risk group, indicating a pronounced association between the risk of DKD progression and elevated mortality rates. Time‐to‐survival probability distribution was displayed in Figure 2A,B.

### 3.3. Association of the Risk of DKD Progression With All‐Cause and Cardiovascular Mortality

In this analysis, we examined the relationships between the risk of DKD progression and mortality rates among individuals with T2D. For all‐cause mortality, the low‐risk group was utilized as the reference group (HR = 1.0). The moderately increased‐risk group showed a 96% higher risk (HR: 1.96; 95% CI: 1.75, 2.20), whereas the high‐risk group exhibited a 244% increase (HR: 3.44; 95% CI: 3.00, 3.95). Notably, the very high‐risk group had an HR of 6.63 (95% CI: 5.75, 7.64), indicating a 563% increased all‐cause mortality risk compared with the low‐risk group. These values remained statistically significant across Models 2–4, particularly for the very high‐risk group, which maintained an HR of 2.53 (95% CI: 2.15, 2.98). Trend analysis confirmed that as DKD progression risk increases, all‐cause mortality risk also rises (*p* for trend < 0.001). For cardiovascular mortality, similar trends were observed. After adjusting for confounding factors, HRs remained clinically significant. In Model 4, the very high‐risk group had a 147% higher cardiovascular mortality risk compared with the low‐risk group (HR: 2.47; 95% CI: 1.87, 3.27). Trend analysis indicated that cardiovascular mortality risk increased with DKD progression risk (*p* for trend < 0.001) (Table 2).

**Table 2:** Hazard ratios associations between the risk stratification of DKD progression and all‐cause and cardiovascular mortality in patients with Type 2 diabetes.

	Number of deaths (*N*)	Model 1	Model 2	Model 3	Model 4
*All-cause mortality*	1781				
Low risk group	724	1.0 (Ref.)	1.0 (Ref.)	1.0 (Ref.)	1.0 (Ref.)
Moderately increased risk group	505	1.96 (1.75, 2.20)	1.54 (1.37, 1.73)	1.43 (1.27, 1.60)	1.39 (1.23, 1.56)
High risk group	284	3.44 (3.00, 3.95)	2.15 (1.87, 2.48)	1.93 (1.67, 2.23)	1.70 (1.47, 1.98)
Very high risk group	268	6.63 (5.75, 7.64)	3.65 (3.15, 4.23)	3.02 (2.59, 3.52)	2.53 (2.15, 2.98)
*p* value for trend		< 0.001	< 0.001	< 0.001	< 0.001
*Cardiovascular mortality*	623				
Low risk group	221	1.0 (Ref.)	1.0 (Ref.)	1.0 (Ref.)	1.0 (Ref.)
Moderately increased risk group	201	2.56 (2.12, 3.10)	1.97 (1.62, 2.40)	1.80 (1.48, 2.20)	1.73 (1.42, 2.12)
High risk group	107	4.26 (3.38, 5.37)	2.55 (2.01, 3.24)	2.22 (1.74, 2.83)	1.93 (1.50, 2.48)
Very high risk group	94	7.66 (6.00, 9.78)	4.07 (3.16, 5.25)	3.02 (2.31, 3.93)	2.47 (1.87, 3.27)
*p* value for trend		< 0.001	< 0.001	< 0.001	< 0.001

*Note:* Cox proportional hazard models were used to estimate hazard ratio (HR) and 95% confidence interval (CI). Model 1, unadjusted. Model 2, adjusted for gender, age, race/ethnicity, family poverty‐income ratio (PIR), education level, marital status. Model 3, Model 2 and further adjusted for alcohol consumption, smoking status, body mass index (BMI), hemoglobin A1c (HbA1c), diabetes duration, history of hypertension, history of cardiovascular disease (CVD), history of stroke, use of glucose‐lowering drugs (including insulin and oral glucose‐lowering drugs), use of renin‐angiotensin system (RAS) inhibitors, and use of lipid‐lowering drugs. Model 4, Model 3 and further adjusted for hemoglobin, total cholesterol, triglycerides, albumin, uric acid.

Abbreviations: DKD, diabetic kidney disease; Ref, reference.

### 3.4. Sensitivity Analyses

Stratified analysis showed that, except for other Hispanic and other race subgroups, both all‐cause and cardiovascular mortality exhibited a significant stepwise increase across ascending risk categories (from low to very high risk, all *p* values for trend < 0.001). Importantly, while the risk gradient remained consistent across gender, BMI, smoking status, metabolic profiles (HbA1c, lipids, and serum UA), and most medication subgroups (all *p* interaction > 0.05), relevant interactions were observed in specific subgroups. Younger patients (< 65 years) exhibited a substantially greater increase in all‐cause mortality upon progression to very high risk (HR: 3.30; 95% CI: 2.32–4.70) compared with older patients (HR: 2.77; 95% CI: 2.29–3.35, *p* interaction = 0.032). Concurrently, RAS inhibitor therapy attenuated the elevated all‐cause mortality risk specifically in moderately increased‐risk patients (users, HR: 1.14; 95% CI: 0.85–1.52 vs. nonusers, HR: 1.54; 95% CI: 1.31–1.81; *p* interaction = 0.003) (Tables S1 and S2).

## 4. Discussion

In this large‐scale national cohort study, we first validated the practical value of the 2024 ADA‐based risk stratification of DKD progression in predicting all‐cause and cardiovascular mortality among patients with T2D. We discovered that the very high‐risk group demonstrated a 1.53‐fold higher all‐cause mortality risk and a 1.47‐fold higher cardiovascular mortality risk compared with the low‐risk group. A pronounced dose‐response gradient was observed, with mortality risk progressively increasing alongside the risk of DKD progression. Notably, this graded relationship remained stable across most examined subgroups, and significant clinical interactions were observed specifically among individuals aged < 65 years and those using RAS inhibitors, suggesting a need for tailored management strategies in clinical practice.

To our knowledge, this study firstly validated the predictive value of 2024 ADA‐based risk stratification matrix of DKD progression for both all‐cause mortality and cardiovascular mortality within a real‐world population with T2D. Previous research by the CKD Prognosis Consortium established the foundation, demonstrating how eGFR and UACR, either individually or combined, predict CKD progression and prognosis [7–10]. This provided the scientific rationale for integrating eGFR and UACR into contemporary risk assessment and CKD staging systems. Subsequent epidemiological studies, including investigations within diabetic cohorts, have consistently affirmed the combined prognostic value of eGFR and UACR. For instance, longitudinal data from 8766 individuals with T2D in the Action in Diabetes and Vascular Disease: Preterax and Diamicron Modified Release Controlled Evaluation Observational (ADVANCE‐ON) study found that independent or combined changes in eGFR and UACR over 2 years predicted major cardiovascular and kidney events [31]. Critically, this study also revealed a significant interaction between eGFR and UACR changes, advocating for nuanced risk assessment [31]. However, ADVANCE‐ON did not specifically evaluate the risk stratification matrix of CKD progression recommended by KDIGO guidelines. Moreover, analysis of 19025 adults in the Hong Kong Diabetes Biobank also employed eGFR and UACR, but focused primarily on comparing adverse outcomes between four DKD phenotypes defined by baseline eGFR and UACR, particularly contrasting nonalbuminuria DKD with other phenotypes [32]—in contrast to our exposure definition (2024 ADA‐based risk stratification matrix of DKD progression). Most recently, a 2025 study of 39,067 patients with Type 1 diabetes from the Swedish National Diabetes Register assessed CKD progression and all‐cause mortality using complex combinations of baseline eGFR and UACR [33]. However, that study excluded stage G5 CKD patients at baseline, utilized different reference groups, and primarily highlighted elevated risks even within early KDIGO categories (no CKD), differing fundamentally from our study objectives and design.

There are a limited number of studies that have directly used guideline‐recommended risk stratification of CKD progression as exposure. A prior study examined the associations between KDIGO‐based risk stratification of DKD progression—using combined eGFR and albuminuria measurements (UACR or dipstick proteinuria)—and all‐cause mortality in US populations, comparing individuals with and without T2D [21]. However, that analysis focused on outcome variations by diabetes status and utilized a nondiabetic low‐risk reference group, distinct from our exclusively diabetic cohort. The Coronary Artery Risk Development in Young Adults (CARDIA) cohort modified the 2012 KDIGO risk heat map of CKD progression into five risk categories ranging from very low to very high risk [22]. Nevertheless, the study primarily focused on the association between risk category progression of CKD progression and the incidence of CVD and all‐cause mortality in young to middle‐aged adults without baseline CVD and the exposure emphasized the dynamic changes in risk categories. Another comprehensive study applied a modified risk stratification of the 2012 KDIGO guidelines to categorize non–dialysis‐dependent CKD patients into five risk strata, similar to the CARDIA cohort [20]. The study found that the achievement rate of cardiovascular risk factor treatment targets sharply declined from 39.7% in the low‐risk group to 16.2% in the very high‐risk group, highlighting substantial disparities in cardiovascular risk management across different risk strata of CKD progression and reinforcing the importance of stratified care [20]. Our study systematically categorized individuals with T2D using the 2024 ADA‐based risk stratification matrix of DKD progression, thoroughly evaluating all‐cause and cardiovascular mortality. Our findings confirm a direct relationship between mortality and risk stratification of DKD progression, validating the predictive power of the 2024 ADA‐recommended stratification approach.

Among the subgroups of “Other Hispanic” and “Other races,” an increasing trend in both all‐cause and cardiovascular mortality risk across higher risk categories of DKD progression was not observed. This absence of a dose–response relationship is likely attributable to the small sample sizes within these demographic categories. Furthermore, younger patients (< 65 years) in our cohort exhibited a disproportionately steeper increase in all‐cause mortality when categorized as very‐high risk. This aligns with findings by Lascar et al. [34], suggesting an elevated complication risk in early‐onset compared with later‐onset T2D. Furthermore, although younger patients had a significantly lower baseline CVD prevalence (*p* < 0.001), they demonstrated significantly poorer glucose control (higher HbA1c), higher low‐density lipoprotein cholesterol (LDL) and TC levels, and a greater proportion with BMI > 30 kg/m^2^ (all *p* < 0.001). These findings, consistent with prior research [35], suggest more effective management of glycemia and cardiovascular risk factors in older individuals with T2D. This age‐specific disparity may also arise because the prognostic significance of frailty and multimorbidity in older individuals possibly outweighs the mortality risk predicted by eGFR and UACR, rendering 2024 ADA‐based risk stratification of DKD progression minimally contributory in this group. Therefore, our results highlight a potential under‐monitoring risk for younger patients and emphasize the need for age‐stratified management strategies. Specifically, developing an “Accelerated Progression Monitoring Protocol” is warranted for high‐risk patients aged < 65 years. Additionally, we observed that RAS inhibitors significantly mitigated mortality risk among moderate‐risk patients, reinforcing the cardiorenal protective value of early RAS blockade. This corroborates findings from a US veteran study showing significantly improved survival with RAS inhibitors therapy in non–dialysis‐dependent CKD patients [36]. These results strongly support early RAS inhibitor intervention in moderate‐risk populations, aligning with guideline recommendations for integrating risk stratification with targeted pharmacotherapy.

This study underscores the practical utility of the 2024 ADA‐based risk stratification system of DKD progression for managing T2D. By leveraging the readily available and clinically feasible combination of eGFR and UACR, our findings enable earlier identification of high‐risk individuals beyond traditional cardiovascular risk models. This facilitates more individualized monitoring and timely intervention, providing practical support for implementing guideline recommendations. Unlike previous studies focusing solely on single biomarkers or complex multimarker algorithms, our approach utilizing the standard risk stratification matrix demonstrates superior real‐world applicability. Integrating this validated risk stratification model into routine clinical workflows holds significant potential to enhance screening efficiency and optimize resource allocation. Future research should investigate whether targeted early intervention within identified high‐risk groups can improve clinical outcomes and modify the disease trajectory.

A key strength of this study is the first validation of the 2024 ADA guideline‐based risk stratification of DKD progression for predicting all‐cause and cardiovascular mortality in a real‐world population with T2D. We achieved this using a comprehensive study design and advanced analytical methods, providing practical evidence supporting the guideline′s clinical implementation. However, there are some limitations in this study. First, residual confounding persists despite rigorous adjustments, inherent to observational designs. Second, risk stratification of DKD progression relied solely on baseline eGFR and UACR, potentially missing their longitudinal fluctuations. Third, generalizability may be limited as NHANES represents the US population, and its ethnic composition and healthcare system differ from Asian or other regions, warranting caution in extrapolation. Fourth, data on newer cardiorenal protective therapies, such as sodium‐glucose cotransporter‐2 (SGLT2) inhibitors, was not available in the dataset and could not be included in the analysis, as the NHANES cycles included in this study (1999–2018) largely predate their widespread clinical use. Therefore, glucose‐lowering therapies were categorized broadly (e.g., insulin or oral agents), which may not fully account for the impact of contemporary treatment strategies on mortality outcomes. Additionally, although NHANES data offers valuable insights, issues of self‐reporting and the risk of biases from long‐term follow‐up highlight the need for further validation through multicenter, prospective large‐sample studies capturing dynamic risk evolution.

## 5. Conclusion

This nationwide study of US adults with T2D confirms the robust prognostic value of the 2024 ADA guideline‐based risk stratification of DKD progression for all‐cause and cardiovascular mortality in real‐world practice. Therefore, incorporating this validated risk stratification system into routine clinical workflows is critical for the timely identification of high‐risk individuals. This facilitates the implementation of targeted surveillance strategies and individualized interventions to improve outcomes.

## Author Contributions

Ying Qiu contributed to conceptualization, data curation, methodology, formal analysis, and writing—original draft. Jun Jiang contributed to investigation and writing—review and editing. Hongqiang Zhang contributed to software and methodology. Jianping Weng contributed to writing—review and editing, as well as supervision. Lei Lan contributed to supervision and funding acquisition. All authors have read and approved the final manuscript.

## Funding

No funding was received for this manuscript.

## Disclosure

All the authors have read and agreed to the published version of the manuscript. The authors have read the STROBE Statement‐checklist of items, and the manuscript was prepared and revised according to the STROBE Statement—checklist of items.

## Ethics Statement

NHANES is conducted by the Centers for Disease Control and Prevention (CDC) and the National Center for Health Statistics (NCHS). The NCHS Research Ethics Review Committee reviewed and approved the NHANES study protocol. All participants signed written informed consent. Additionally, this study was also reviewed and approved by the Ethics Committee of the First Affiliated Hospital of the University of Science and Technology of China (ID: 2026‐ky180).

## Conflicts of Interest

The authors declare no conflicts of interest.

## Supporting information


**Supporting Information** Additional supporting information can be found online in the Supporting Information section. Supporting Information. Table S1 Stratified analyses of the associations between the risk stratification of DKD progression and all‐cause mortality in patients with Type 2 diabetes. Supporting Information. Table S2 Stratified analyses of the associations between the risk stratification of DKD progression and cardiovascular mortality in patients with Type 2 diabetes.

## Data Availability

The data that support the findings of this study are openly available in National Health and Nutrition Examination Survey (NHANES) at https://www.cdc.gov/nchs/nhanes/?CDC_AAref_Val=https://www.cdc.gov/nchs/nhanes/index.htm.
