# Supplementary material for: Risk Stratification for Diabetic Kidney Disease Progression in Predicting All‐Cause and Cardiovascular Mortality in Type 2 Diabetes: A National Cohort Study From NHANES 1999–2018
Source: J Diabetes Res. 2026 Jun 4;2026:9563077. doi: 10.1155/jdr/9563077 (PMC13581058; doi:10.1155/jdr/9563077)
Supplement: Supplementary file 1 [file JDR-2026-9563077-s001.doc]

### **Supplementary material files**

**Supplementary Table 1** Stratified analyses of the associations between the risk stratification of DKD progression and all-cause mortality in patients with type 2 diabetes

| **Characteristics** | **N** | **Low risk group** | **Moderately increased risk group** | **High risk group** | **Very high risk group** | ***p* value for trend** | ***p* value for interaction** |
| --- | --- | --- | --- | --- | --- | --- | --- |
| **Age (years)** |  |  |  |  |  |  | **0.032** |
| <65 | 4101 | 1.0(Ref.) | 1.42 (1.15, 1.75) | 1.67 (1.25, 2.21) | 3.30 (2.32, 4.70) | <0.001 |  |
| ≥65 | 2835 | 1.0(Ref.) | 1.53 (1.33, 1.77) | 1.87 (1.56, 2.23) | 2.77 (2.29, 3.35) | <0.001 |  |
| **Gender** |  |  |  |  |  |  | 0.248 |
| Male | 3601 | 1.0(Ref.) | 1.38 (1.18, 1.62) | 1.74 (1.43, 2.12) | 2.23 (1.79, 2.78) | <0.001 |  |
| Female | 3335 | 1.0(Ref.) | 1.37 (1.14, 1.65) | 1.64 (1.29, 2.07) | 3.08 (2.38, 3.98) | <0.001 |  |
| **Race/ethnicity** |  |  |  |  |  |  | 0.401 |
| Mexican American | 1529 | 1.0(Ref.) | 1.31 (1.03, 1.66) | 1.64 (1.21, 2.21) | 3.56 (2.59, 4.89) | <0.001 |  |
| Other Hispanic | 697 | 1.0(Ref.) | 1.90 (1.15, 3.14) | 1.02 (0.50, 2.07) | 1.55 (0.67, 3.59) | 0.311 |  |
| Non-Hispanic White | 2279 | 1.0(Ref.) | 1.46 (1.22, 1.74) | 1.79 (1.45, 2.22) | 2.49 (1.93, 3.21) | <0.001 |  |
| Non-Hispanic Black | 1747 | 1.0(Ref.) | 1.21 (0.94, 1.56) | 1.69 (1.22, 2.35) | 2.62 (1.92, 3.58) | <0.001 |  |
| Other Race | 684 | 1.0(Ref.) | 1.82 (0.96, 3.45) | 1.94 (0.79, 4.73) | 1.92 (0.73, 5.00) | 0.105 |  |
| **BMI (kg/m^2^)** |  |  |  |  |  |  | 0.666 |
| <30 | 3082 | 1.0(Ref.) | 1.46 (1.24, 1.72) | 1.72 (1.40, 2.11) | 2.69 (2.14, 3.38) | <0.001 |  |
| ≥30 | 3854 | 1.0(Ref.) | 1.32 (1.11, 1.57) | 1.69 (1.36, 2.10) | 2.43 (1.91, 3.09) | <0.001 |  |
| **Smoking status** |  |  |  |  |  |  | 0.689 |
| Never | 3502 | 1.0(Ref.) | 1.39 (1.15, 1.69) | 1.73 (1.37, 2.20) | 2.53 (1.96, 3.25) | <0.001 |  |
| Former | 2247 | 1.0(Ref.) | 1.30 (1.07, 1.57) | 1.43 (1.14, 1.81) | 2.39 (1.84, 3.11) | <0.001 |  |
| Current | 1187 | 1.0(Ref.) | 1.50 (1.14, 1.98) | 2.22 (1.51, 3.27) | 3.55 (2.22, 5.70) | <0.001 |  |
| **HbA1c (%)** |  |  |  |  |  |  | 0.908 |
| <7.0 | 3821 | 1.0(Ref.) | 1.36 (1.16, 1.60) | 1.65 (1.34, 2.02) | 2.58 (2.06, 3.23) | <0.001 |  |
| ≥7.0 | 3115 | 1.0(Ref.) | 1.44 (1.21, 1.72) | 1.80 (1.45, 2.23) | 2.63 (2.07, 3.36) | <0.001 |  |
| **Total cholesterol (mmol/L)** |  |  |  |  |  |  | 0.599 |
| ≤5.18 | 4288 | 1.0(Ref.) | 1.39 (1.19, 1.63) | 1.67 (1.38, 2.01) | 2.71 (2.21, 3.33) | <0.001 |  |
| >5.18 | 2648 | 1.0(Ref.) | 1.36 (1.12, 1.64) | 1.80 (1.40, 2.30) | 2.32 (1.75, 3.06) | <0.001 |  |
| **Hyperuricemia** |  |  |  |  |  |  | 0.865 |
| No | 5125 | 1.0(Ref.) | 1.40 (1.22, 1.61) | 1.77 (1.47, 2.13) | 2.59 (2.09, 3.22) | <0.001 |  |
| Yes | 1811 | 1.0(Ref.) | 1.41 (1.12, 1.79) | 1.84 (1.43, 2.38) | 3.07 (2.35, 4.02) | <0.001 |  |
| **Hypertention** |  |  |  |  |  |  | 0.116 |
| No | 1527 | 1.0(Ref.) | 1.65 (1.14, 2.40) | 2.22 (1.20, 4.11) | 4.03 (1.62, 10.05) | <0.001 |  |
| Yes | 5409 | 1.0(Ref.) | 1.35 (1.19, 1.53) | 1.67 (1.43, 1.95) | 2.48 (2.09, 2.93) | <0.001 |  |
| **Use of glucose-lowering drugs** |  |  |  |  |  |  | 0.177 |
| No | 2759 | 1.0(Ref.) | 1.47 (1.22, 1.78) | 1.61 (1.24, 2.10) | 3.15 (2.37, 4.18) | <0.001 |  |
| Yes | 4177 | 1.0(Ref.) | 1.37 (1.17, 1.60) | 1.76 (1.46, 2.12) | 2.35 (1.91, 2.88) | <0.001 |  |
| **Use of RAS inhibitors** |  |  |  |  |  |  | **0.003** |
| No | 2858 | 1.0(Ref.) | 1.54 (1.31, 1.81) | 1.73 (1.42, 2.10) | 2.58 (2.10, 3.17) | <0.001 |  |
| Yes | 1552 | 1.0(Ref.) | 1.14 (0.85, 1.52) | 2.03 (1.43, 2.88) | 2.30 (1.50, 3.52) | <0.001 |  |
| **Use of lipid-lowering drugs** |  |  |  |  |  |  | 0.132 |
| No | 3852 | 1.0(Ref.) | 1.37 (1.17, 1.61) | 1.69 (1.37, 2.08) | 2.73 (2.15, 3.46) | <0.001 |  |
| Yes | 3077 | 1.0(Ref.) | 1.41 (1.18, 1.69) | 1.67 (1.35, 2.08) | 2.43 (1.93, 3.07) | <0.001 |  |

DKD, diabetic kidney disease; Ref, reference

Cox proportional hazard models were used to estimate hazard ratio (HR) and 95% confidence interval (CI) after adjusting for gender, age, race/ethnicity, family poverty-income ratio (PIR), education level, marital status, alcohol consumption, smoking status, body mass index (BMI), history of hypertension, history of cardiovascular disease (CVD), history of stroke, use of glucose-lowering drugs (including insulin and oral glucose-lowering drugs), use of renin-angiotensin system (RAS) inhibitors and use of lipid-lowering drugs, hemoglobin, hemoglobin A1c (HbA1c), total cholesterol, triglycerides, albumin, uric acid, but the model was not adjusted for the stratification variable itself.

**Supplementary Table 2** Stratified analyses of the associations between the risk stratification of DKD progression and cardiovascular mortality in patients with type 2 diabetes

| **Characteristics** | **N** | **Low risk group** | **Moderately increased risk group** | **High risk group** | **Very high risk group** | ***p* value for trend** | ***p* value for interaction** |
| --- | --- | --- | --- | --- | --- | --- | --- |
| **Age (years)** |  |  |  |  |  |  | 0.105 |
| <65 | 4101 | 1.0(Ref.) | 1.85 (1.29, 2.66) | 1.67 (1.01, 2.77) | 4.26 (2.34, 7.77) | <0.001 |  |
| ≥65 | 2835 | 1.0(Ref.) | 1.87 (1.47, 2.38) | 2.10 (1.57, 2.81) | 2.39 (1.72, 3.32) | <0.001 |  |
| **Gender** |  |  |  |  |  |  | 0.221 |
| Male | 3601 | 1.0(Ref.) | 1.72 (1.32, 2.25) | 2.18 (1.58, 3.00) | 2.44 (1.69, 3.53) | <0.001 |  |
| Female | 3335 | 1.0(Ref.) | 1.70 (1.25, 2.31) | 1.49 (0.98, 2.26) | 2.50 (1.59, 3.93) | <0.001 |  |
| **Race/ethnicity** |  |  |  |  |  |  | 0.625 |
| Mexican American | 1529 | 1.0(Ref.) | 1.80 (1.10, 2.94) | 1.63 (0.83, 3.21) | 3.36 (1.55, 7.31) | 0.003 |  |
| Other Hispanic | 697 | 1.0(Ref.) | 3.48 (1.33, 9.11) | 0.76 (0.18, 3.25) | 2.10 (0.44, 10.13) | 0.610 |  |
| Non-Hispanic White | 2279 | 1.0(Ref.) | 1.70 (1.26, 2.29) | 2.12 (1.49, 3.00) | 2.51 (1.65, 3.82) | <0.001 |  |
| Non-Hispanic Black | 1747 | 1.0(Ref.) | 1.49 (0.99, 2.26) | 1.91 (1.10, 3.30) | 2.63 (1.53, 4.53) | <0.001 |  |
| Other Race | 684 | 1.0(Ref.) | 1.34 (0.53, 3.40) | 0.24 (0.07, 0.83) | 3.78 (1.37, 10.47) | 0.564 |  |
| **BMI (kg/m^2^)** |  |  |  |  |  |  | 0.804 |
| <30 | 3082 | 1.0 (Ref.) | 1.71 (1.29, 2.26) | 1.70 (1.19, 2.43) | 2.19 (1.47, 3.28) | <0.001 |  |
| ≥30 | 3854 | 1.00(Ref.) | 1.77 (1.31, 2.37) | 2.18 (1.52, 3.12) | 2.74 (1.83, 4.10) | <0.001 |  |
| **Smoking status** |  |  |  |  |  |  | 0.065 |
| Never | 3502 | 1.0(Ref.) | 1.67 (1.23, 2.26) | 1.54 (1.04, 2.28) | 2.03 (1.35, 3.06) | <0.001 |  |
| Former | 2247 | 1.0(Ref.) | 1.64 (1.17, 2.28) | 1.77 (1.19, 2.64) | 2.38 (1.46, 3.88) | <0.001 |  |
| Current | 1187 | 1.0(Ref.) | 2.02 (1.21, 3.37) | 3.41 (1.79, 6.49) | 5.80 (2.64, 12.74) | <0.001 |  |
| **HbA1c (%)** |  |  |  |  |  |  | 0.767 |
| <7.0 | 3821 | 1.0(Ref.) | 1.49 (1.14, 1.96) | 1.63 (1.15, 2.30) | 2.27 (1.55, 3.33) | <0.001 |  |
| ≥7.0 | 3115 | 1.0(Ref.) | 2.02 (1.49, 2.73) | 2.19 (1.51, 3.16) | 2.67 (1.75, 4.08) | <0.001 |  |
| **Total cholesterol (mmol/L)** |  |  |  |  |  |  | 0.220 |
| ≤5.18 | 4288 | 1.0(Ref.) | 1.66 (1.27, 2.16) | 1.72 (1.24, 2.38) | 2.84 (2.00, 4.04) | <0.001 |  |
| >5.18 | 2648 | 1.0(Ref.) | 1.77 (1.29, 2.43) | 2.47 (1.64, 3.71) | 2.13 (1.30, 3.47) | <0.001 |  |
| **Hyperuricemia** |  |  |  |  |  |  | 0.902 |
| No | 5125 | 1.0(Ref.) | 1.78 (1.40, 2.25) | 2.11 (1.53, 2.90) | 2.57 (1.76, 3.77) | <0.001 |  |
| Yes | 1811 | 1.0(Ref.) | 1.50 (1.08, 2.09) | 1.89 (1.34, 2.67) | 4.42 (3.33, 5.86) | <0.001 |  |
| **Hypertention** |  |  |  |  |  |  | 0.332 |
| No | 1527 | 1.0(Ref.) | 2.92 (1.38, 6.19) | 3.01 (0.83, 10.90) | 2.36 (0.40, 14.02) | 0.020 |  |
| Yes | 5409 | 1.0(Ref.) | 1.64 (1.33, 2.02) | 1.86 (1.44, 2.40) | 2.35 (1.77, 3.13) | <0.001 |  |
| **Use of glucose-lowering drugs** |  |  |  |  |  |  | 0.265 |
| No | 2759 | 1.0(Ref.) | 1.98 (1.43, 2.73) | 1.75 (1.10, 2.80) | 3.39 (2.06, 5.58) | <0.001 |  |
| Yes | 4177 | 1.0(Ref.) | 1.62 (1.25, 2.10) | 1.92 (1.42, 2.60) | 2.02 (1.42, 2.86) | <0.001 |  |
| **Use of RAS inhibitors** |  |  |  |  |  |  | 0.081 |
| No | 2858 | 1.0(Ref.) | 1.68 (1.29, 2.19) | 1.88 (1.37, 2.58) | 2.34 (1.67, 3.28) | <0.001 |  |
| Yes | 1552 | 1.0(Ref.) | 1.61 (1.00, 2.61) | 2.22 (1.19, 4.16) | 1.98 (0.90, 4.34) | 0.009 |  |
| **Use of lipid-lowering drugs** |  |  |  |  |  |  | 0.175 |
| No | 3852 | 1.0(Ref.) | 1.96 (1.47, 2.60) | 2.47 (1.73, 3.51) | 2.79 (1.84, 4.22) | <0.001 |  |
| Yes | 3077 | 1.0(Ref.) | 1.57 (1.17, 2.10) | 1.49 (1.04, 2.15) | 2.07 (1.40, 3.06) | <0.001 |  |

DKD, diabetic kidney disease; Ref, reference

Cox proportional hazard models were used to estimate hazard ratio (HR) and 95% confidence interval (CI) after adjusting for gender, age, race/ethnicity, family poverty-income ratio (PIR), education level, marital status, alcohol consumption, smoking status, body mass index (BMI), history of hypertension, history of cardiovascular disease (CVD), history of stroke, use of glucose-lowering drugs (including insulin and oral glucose-lowering drugs), use of renin-angiotensin system (RAS) inhibitors and use of lipid-lowering drugs, hemoglobin, hemoglobin A1c (HbA1c), total cholesterol, triglycerides, albumin, uric acid, but the model was not adjusted for the stratification variable itself.
